# microRNA-29b mediates fibrotic induction of human xylosyltransferase-I in human dermal fibroblasts via the Sp1 pathway

**DOI:** 10.1038/s41598-018-36217-2

**Published:** 2018-12-12

**Authors:** Lara Riedel, Bastian Fischer, Thanh-Diep Ly, Doris Hendig, Joachim Kuhn, Cornelius Knabbe, Isabel Faust

**Affiliations:** Institut für Laboratoriums- und Transfusionsmedizin, Herz- und Diabeteszentrum Nordrhein-Westfalen, Universitätsklinik der Ruhr-Universität Bochum, Georgstraße 11, 32545 Bad Oeynhausen, Germany

## Abstract

Diminished microRNA-29b levels have recently been revealed to provoke increased expression and accumulation of extracellular matrix molecules, such as collagens in fibrotic remodeling. Subsequently, the aim of this study was to find out whether microRNA-29b might also regulate human xylosyltransferase (XT)-I expression. XT-I has been characterized previously as a fibrosis biomarker catalyzing the key step of proteoglycan biosynthesis. While we demonstrate that *XYLT1* is neither a target of microRNA-29b identified *in silico* nor a direct 3′ untranslated region binding partner of microRNA-29b, transfection of normal human dermal fibroblasts with microRNA-29b inhibitor strongly increased *XYLT1* mRNA expression and XT activity. Combined results of the target prediction analysis and additional transfection experiments pointed out that microRNA-29b exerts indirect influence on XT-I by targeting the transcription factor specificity protein 1 (Sp1). We could confirm our hypothesis due to the decrease in *XYLT1* promoter activity after Sp1 binding site mutation and the approval of occupancy of these binding sites by Sp1 *in vitro*. Taken together, a hitherto unidentified pathway of XT-I regulation via microRNA-29b/Sp1 was determined in this study. Our observations will facilitate the understanding of complex molecular fibrotic pathways and provide new opportunities to investigate microRNA-based antifibrotic tools.

## Introduction

MicroRNAs (miRNAs) are small endogenous noncoding RNA molecules, which consist of 18 to 25 nucleotides. Together with an RNA-induced silencing protein complex, a miRNA interacts with the 3′ untranslated region (3′UTR) of its target mRNAs. In the case of perfect base pairing between the miRNA and target sequence, the mRNA is degraded. Contrarily, a repression of translation takes place in the case of imperfect base pairing. Therefore, miRNAs represent a cellular tool for posttranscriptional regulation^1,2^.

As published earlier, 1 to 5% of the human genome code for miRNAs, whereby each miRNA can bind several target mRNAs^3^. Many diseases have been associated with abnormal miRNA expression profiles, so that miRNAs are not only used as biomarkers, but also handled as potential therapeutic targets to exert influence on dysregulated molecular pathways^4^. Several miRNA mimics or antimiRs have currently reached clinical trials, whereby recent advances in technologies to deliver miRNAs *in vivo* will assure a safe and targeted application of miRNA therapeutics in the future^5^.

One type of disease associated with abnormal miRNA expression is fibrosis^6,7^. Fibrotic remodeling occurs in different tissues and is characterized by an excessive cellular synthesis of extracellular matrix (ECM) molecules promoting scar tissue formation. Although many underlying molecular processes are well described to date, a therapeutic approach has not yet been established. Among others, current studies deal with the differentiation of fibroblasts to matrix synthesizing contractile myofibroblasts, which were identified as the key mediator cells of fibrotization. In comparison to physiological wound healing processes, myofibroblasts in fibrotic tissues do not become apoptotic after scar tissue formation but persist producing ECM due to the uninterrupted stimulation with fibrotic cytokines, such as transforming growth factor-β1 (TGF-β1). Consequently, an imbalance of ECM synthesis promotes tissue stiffening, damage and functional organ deficit^8,9^.

It has been revealed recently that several miRNAs, such as miRNA-21, miRNA-145 or miRNA-29b, are expressed aberrantly in fibrotic diseases. Zhou *et al*.^10^ found that the miRNA-21 level in the serum of systemic sclerosis (SSc) is highly induced, while they identified 21 miRNAs which are differentially expressed in SSc skin tissues. In line with these observations, an attenuation of unilateral ureteral obstruction-induced renal fibrosis after blocking miRNA-21 *in vivo* was reported^11^. miRNA-145 expression was demonstrated to be upregulated in the tissue of idiopathic pulmonary fibrosis patients and in lung fibroblasts treated with TGF-β1. A miRNA-145 deficiency was further evaluated to be protective against bleomycin-induced lung fibrosis in a mouse model^12^.

In this study, we focus on miRNA-29b, which is strongly downregulated in various fibrotic tissues^13–15^. The miRNA-29 family decreases the expression of collagens and other ECM components directly; consequently, these are increased in fibrotic tissues, such as skin sections of SSc patients^16^. miRNA-29b was also shown to be diminished in SSc serum exosomes, which is why miRNA-29b was recently defined as a key regulator of SSc^17^.

The challenge in developing new anti-fibrotic tools is to understand the underlying molecular pathways of fibrotization and translate these molecular discoveries into targeted therapeutic drugs^18^. One putative downstream target of miRNA-29b we consider here is human xylosyltransferase (XT)-I (EC 2.4.2.26). XT-I is one of two human isoenzymes which catalyzes the rate-limiting step of proteoglycan (PG) glycosylation by transferring UDP-xylose to defined serine residues of a PG core protein^19,20^. Subsequent sugars are added by other glycosyltransferases, resulting finally in the formation of glycosaminoglycans^21^. PG and their linked glycosaminoglycan residues define, together with collagens and other structural components, the architecture of the ECM and are, therefore, involved in a wide range of cellular functions, including proliferation, signaling, development and adhesion^22^. Since XT are shed from the golgi membrane in the extracellular space, XT activity is not only the pacemaker of PG synthesis, but also a suitable fibrosis biomarker. Due to the shedding, 90% of XT activity is found in cell culture supernatants and only a minority of the enzyme remains in the cellular membrane. Thus XT activity quantified in cell culture supernatants is a useful link to *XYLT* mRNA expression quantified in cell lysates. However, at present it remains unclear whether an increase in XT activity is solely caused by increased translation and protein expression or whether other mechanisms as for instance mRNA stability or posttranslational modifications are involved, too.

Quantification of serum XT activity of liver fibrosis or SSc patients revealed a significantly increased XT activity, which goes along with the upregulated PG synthesis in fibrotic tissues^23,24^. On top of that, TGF-β1-induced upregulated *XYLT1* mRNA expression and XT activity are associated with dermal myofibroblast differentiation and, therefore, qualify XT as an excellent cellular fibrosis biomarker similar to alpha smooth muscle actin expression^25^. Surprisingly, the coexistence of two XT isoenzymes has not yet been fully elucidated. In addition to several biochemical property differences described earlier, we only identified XT-I, but not XT-II, as being involved in fibrotic remodeling. However, we assume that XT-I expression is not only inducible by TGF-β1, but also might be regulated by hitherto unidentified molecular pathways providing the opportunity for therapeutic intervention.

Regarding this, no description of a XT-I regulation by miRNAs has been described or postulated to date. Therefore, the aim of this study was to find out whether one of the most important fibrosis-associated miRNAs, miRNA-29b, exerts influence on *XYLT1* mRNA expression. We used normal human dermal fibroblasts (NHDF) of healthy controls, which have been shown previously to be an adequate cellular model system for the investigation of molecular processes in dermal fibrosis, to evaluate this issue. The results of our study reveal for the first time that XT-I is indirectly upregulated via the miRNA-29b/Sp1 pathway in fibrosis.

## Results

### TGF-β1 supplementation downregulates miRNA-29b expression in NHDF

**Figure 1:**
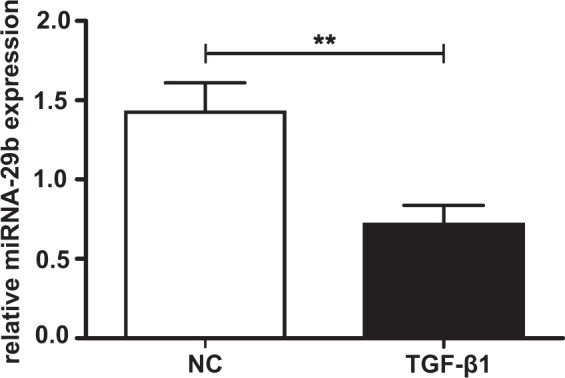
Effect of TGF-β1 supplementation on miRNA-29b expression in NHDF. NHDF cell lines (n = 2) were seeded (50 cells/mm²) and incubated for 24 h. Thereafter, a serum withdrawal (0.1% FCS) lasting 24 h was performed. Finally, the cell culture medium was supplemented with TGF-β1 (5 ng/mL) or PBS (negative control, NC), respectively. After 48 h, miRNA-29b expression was quantified by using Taqman probes in a quantitative real-time PCR. miRNA-29b expression was normalized to the appropriate expression level of the housekeeping gene miRNA-191 and is expressed as a ratio to one cell line. Data are presented as means +/− SEM with corresponding standard error for three biological replicates per cell line. **p < 0.01 (Mann-Whitney U test).

According to the literature, miRNA-29b expression is downregulated in various cell culture models after fibrotic supplementation of TGF-β1^26^. Gene expression analysis using a miRNA PCR array was performed to verify this in our myofibroblast cultivation system. We found that miRNA-29b expression was 0.4-fold diminished in NHDF treated with TGF-β1 compared to controls (data not shown). This result was confirmed by a Taqman-based real-time PCR assay, which revealed a 0.5-fold reduction of miRNA-29b expression in NHDF treated with TGF-β1 compared to untreated controls (Fig. 1). Thus, our cell culture system represents a reliable experimental setting to study a putative linkage between TGF-β1-induced miRNA-29b downregulation and TGF-β1-induced XT-I upregulation. The latter has been described recently^25^.

### *In silico* analysis depicts a miRNA-29b-mediated mRNA expression regulation of various fibrosis-associated genes, except *XYLT1*

Since fibrotic tissue remodeling is accompanied by TGF-β1-induced changes in miRNA-29b and *XYLT1* expression, our aim was to investigate whether *XYLT1* might be regulated directly by miRNA-29b. This was evaluated by analyzing the predicted targets of miRNA-29b *in silico*. The comparison of results of three independent databases indicated several congruent target mRNAs (Table 1). Due to the high number of putative targets predicted by the software tools, a manual selection of ECM- and fibrosis-related targets was accomplished. All of the targets listed in Table 1 have been associated with fibrotic remodeling previously, such as collagens, fibrillin 1 and transcription factors (Sp1 and Krueppel-like factors)^27,28^. Remarkably, none of the *in silico* analyses predicted *XYLT1* mRNA to be a target of miRNA-29b. However, it was shown earlier that the *XYLT1* promoter region harbors several Sp1 binding sites^29,30^. Thus, a putative *XYLT1* regulation via the miRNA-29b/Sp1 pathway had to be elucidated.

**Table 1:** List of targets of miRNA-29b identified *in silico*.

Target	PicTar	TargetScan	DIANA mT
*COL11A1*	17.93	−1.24	0.98924
*COL1A1*	10.92		0.96684
*COL3A1*	8.24	−1.71	0.99989
*COL4A1*	10.03	−0.70	0.99999
*COL5A2*	7.07		0.98480
*COL7A1*	7.47	−0.59	0.99990
*FBN1*	8.23		
*KLF13*	17.00		
*SP1*	15.00	−0.18	

Three independent software tools (Target Scan, PicTar and DIANA mT) were used to identify the targets of miRNA-29b. A manual selection of ECM- or fibrosis-related targets is listed (left column: *COL11A1* collagen type 11 α 1, *COL1A1* collagen type 1 α 1, *COL3A1* collagen type 3 α 1, *COL4A1* collagen type 4 α 1, *COL5A1* collagen type 5 α 1, *COL7A1* collagen type 7 α 1, *FBN1* fibrillin 1, *KLF13* Krueppel-like factor 13, *SP1* spec ificity protein 1. Each target prediction tool uses its own mathematical algorithm which describes the binding intensity of miRNA to mRNA in terms of an indicated score^63–65^. Concerning Target Scan, a more negative score represents the best binding conditions, while a high score of PicTar and DIANA mT suggests strong miRNA binding to the appropriate mRNA.

### miRNA-29b does not regulate *XYLT1* mRNA expression via binding to the *XYLT1* 3′UTR

**Figure 2:**
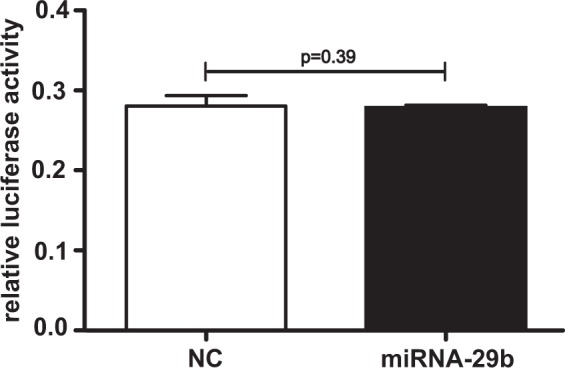
Evaluation of direct miRNA-29b binding to the *XYLT1* 3′UTR. SW1353 cells were seeded and incubated for 24 h. Cells were transfected with *XYLT1* 3′UTR coding luciferase vectors and miRNA-29b or a negative control miRNA (NC). Cell lysates were harvested after 48 h, and firefly luciferase activity and renilla luciferase activity were determined. Changes in relative luciferase activity resemble the putative interaction of miRNA-29b with the 3′UTR of *XYLT1* mRNA. Results are expressed as means +/− SEM for six biological replicates (Mann-Whitney U test).

SW1353 chondrosarcoma cells were transfected with miRNA-29b and luciferase plasmids coding for the 3′UTR of *XYLT1* to further examine whether miRNA-29b binds directly to the 3′UTR of *XYLT1 in vitro*. Quantified firefly luciferase activity, which is influenced by miRNA-29b binding to the *XYLT1* 3′UTR, was normalized to constantly expressed renilla luciferase activity. By comparing the relative luciferase activity of cell lysates transfected with miRNA-29b or a negative control miRNA, it could be demonstrated that miRNA-29b transfection did not exert any influence on relative luciferase activity. Thus, miRNA-29b does not bind directly to the *XYLT1* 3′UTR (Fig. 2).

### *XYLT1* mRNA expression and XT activity are regulated indirectly by miRNA-29b

**Figure 3:**
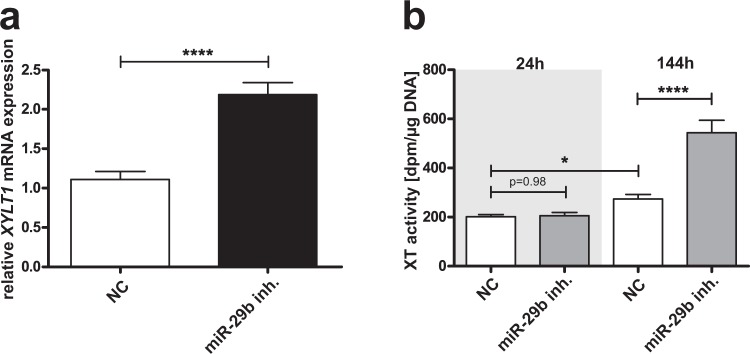
Influence of miRNA-29b inhibitor on the *XYLT1* mRNA expression level and XT activity in NHDF. NHDF cell lines (n = 2) were seeded and transfected with miRNA-29b inhibitor (miR-29b inh.) or a control miRNA (NC). The relative *XYLT1* mRNA expression level was determined by quantitative real-time PCR after 48 h. Data were normalized to a normalization factor calculated from the geometric mean of *HPRT*, *GAPDH* and *B2M* mRNA expression levels. The values indicated are expressed as ratio to one cell line (**a**). The XT activity (**b**) was quantified after 48 and 144 h in cell culture supernatants by using a radiochemical enzymatic assay. The XT activity in dpm was referred to the DNA content of the appropriate cell lysate. Values are means +/− SEM for three biological replicates per cell line; *p < 0.05; ****p < 0.0001 (Mann-Whitney U test).

Although no direct binding of miRNA-29b to the *XYLT1* mRNA 3′UTR could be observed, we analyzed whether an indirect regulation might participate in abnormal XT regulation in fibrosis. We conducted several cell culture experiments to determine this. At first, NHDF were transfected with a miRNA-29b inhibitor to resemble the miRNA suppression in fibrosis. *XYLT1* mRNA expression was 2.0-fold increased after treatment in comparison to controls (NC, Fig. 3a). In accordance with the changes in *XYLT1* mRNA expression detected, XT activity was also 2.0-fold increased in cell culture supernatants after 144 h of miRNA-29b inhibitor treatment compared to controls (Fig. 3b). No difference in XT activity between treated and untreated cells could be detected after 48 h. A slight 1.4-fold time-dependent increase in the XT activity of the controls originated from enzyme accumulation in the cell culture supernatant.

**Figure 4:**
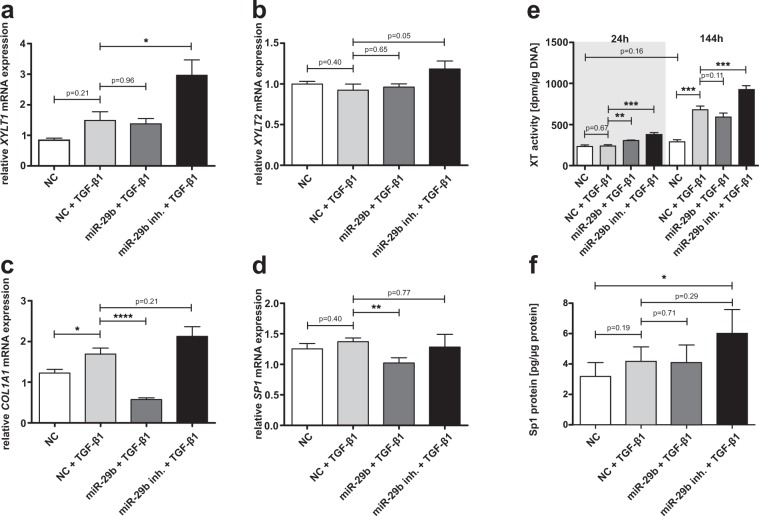
Influence of the combinatory effect of miRNA-29b or miRNA-29b inhibitor with TGF-β1 on NHDF mRNA expression levels, XT activity and Sp1 protein expression. NHDF cell lines (n = 2) were seeded and after 24 h, transfection with a negative control miRNA (NC), miRNA-29b (miR-29b) or its inhibitor (miR-29b inh.) was performed. The cell culture medium was changed 6 h later and supplemented with TGF-β1 or vehicle (NC), as indicated. The cells were harvested 24 h after TGF-β1 supplementation for relative quantification of the mRNA expression levels of *XYLT1* (**a**), *XYLT2* (**b**), *COL1A1* (**c**) and *SP1* (**d**). Data were normalized to a normalization factor, determined by calculating the geometric mean of *HPRT*, *GAPDH* and *B2M* mRNA expression levels, and expressed as a ratio to one cell line. The XT activity (**e**) was quantified after 24 and 144 h in cell culture supernatants by using a radiochemical enzymatic assay. The XT activity in dpm was referred to the DNA content of the appropriate cell lysate. The Sp1 protein concentration (**f**) was measured in nuclear extracts via ELISA and referred to total nuclear protein content. Values are means ± SEM for three biological replicates per cell line *p < 0.05; **p < 0.01; ***p < 0.001; ****p < 0.0001 (Mann-Whitney U-test).

In a second experimental setting, we analyzed *XYLT1* (Fig. 4a), *XYLT2* (Fig. 4b), *COL1A1* (Fig. 4c) and *SP1* (Fig. 4d) mRNA expression levels as well as XT activity (Fig. 4e) and Sp1 protein content (Fig. 4f) after treating NHDF with miRNA-29b or its inhibitor under supplementation of TGF-β1 or a vehicle, respectively. It is possible using this approach to figure out any regulatory effects of miRNA-29b or its inhibitor which might strengthen or weaken the regulatory effect of TGF-β1 compared with a fibrotic state.

*XYLT1* mRNA expression showed a slight 1.8-fold but not significant increase after supplementation of TGF-β1 compared to untreated cells. An additional supply of miRNA-29b did not exert any influence, but transfection with miRNA-29b inhibitor resulted in a significant 2.0-fold upregulation of *XYLT1* mRNA expression. *XYLT2* mRNA expression was not influenced by any treatment. Incubation of NHDF with TGF-β1 resulted in a significantly 1.4-fold upregulated *COL1A1* mRNA expression level. TGF-β1 supplementation in parallel with miRNA-29b or miRNA-29b inhibitor transfection entailed a strong 0.3-fold reduction or a slight but not significant increase of the *COL1A1* mRNA expression, respectively. Determination of *SP1* mRNA expression only depicted a 0.8-fold downregulation after miRNA-29b transfection under supplementation of TGF-β1. Treatment solely with TGF-β1 or combined incubation with TGF-β1 and miRNA-29b inhibitor did not affect *SP1* mRNA expression levels significantly. Changes in the XT activity resembled the effects observed in the *XYLT1* mRNA expression level. Differences between the treatment groups were more pronounced after 144 h, because XT accumulate in the cell culture supernatant. We detected a strong 2.3-fold increase in XT activity after cultivating NHDF in the presence of TGF-β1 compared to controls. Additional transfection with miRNA-29b or its inhibitor was found to result in a slight but not significant decrease or a 1.4-fold significant induction of XT activity, respectively.

An ELISA was performed to determine differences in the Sp1 protein level. TGF-β1 treatment induced Sp1 protein content tendentially, but not significantly. Combined supplementation of TGF-β1 and miRNA-29b inhibitor resulted in a significant 1.9-fold increase of the Sp1 protein concentration in comparison to untreated cells. According to our hypothesis, this observation suggests an involvement of Sp1 in the miRNA-29b-mediated *XYLT1* upregulation. It has been shown previously that Sp1 plays a crucial role in *XYLT1* regulation and that miRNA-29b targets Sp1 in a fibrotic context^30,31^. However, we addressed the relevance of Sp1 transcription factor binding sites for *XYLT1* expression and the occupancy of these transcription factor binding sites *in vitro* to further support our hypothesis of a fibrotic interplay of miRNA-29b, Sp1 and XT-I.

### *XYLT1* promoter activity is strongly influenced by the deletion of Sp1 transcription factor binding sites

**Figure 5:**
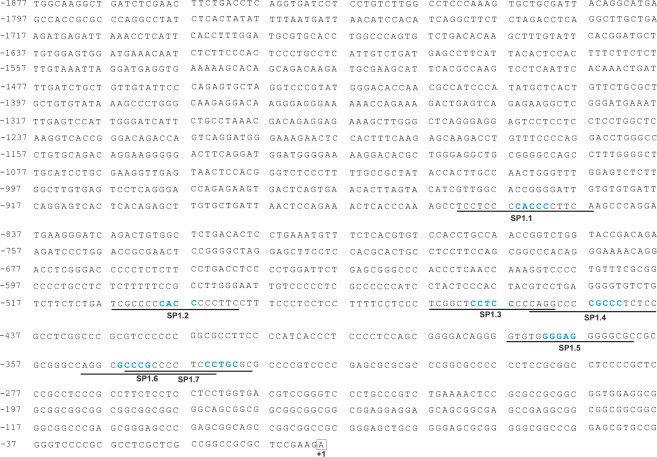
Complete *XYLT1* promoter sequence containing the Sp1 transcription factor binding sites identified. The genomic DNA sequence of the human *XYLT1* promoter region (GenBank Accession Number KM079589 and NG_015843.1) has been described recently^29^. Numbers on the left define the nucleotide position up- or downstream according to the translation initiation site ATG (c. +1) indicated in bold. Sp1 transcription factor binding sites identified *in silico* (named SP1.1 to SP1.7) are underlined, while nucleotides highlighted blue were modified by site-directed mutagenesis to delete the transcription factor binding site.

**Figure 6:**
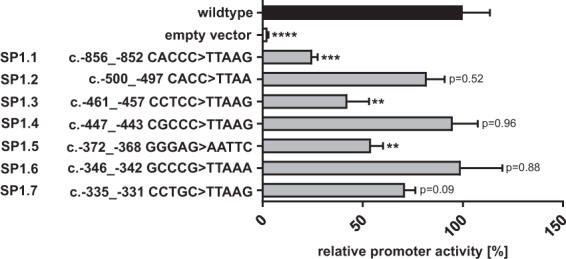
Quantification of *XYLT1* promoter activity after site-directed mutagenesis of Sp1 transcription factor binding sites. The *XYLT1* wildtype vector (pGL4.10 *XYLT1* c. − 1639 to c. + 1_complete_)^29^ was used to delete Sp1 transcription factor binding sites by site-directed mutagenesis. Modified sequences are indicated on the left. SW1353 cells were transfected with the promoter plasmids and relative promoter activity was quantified by dual luciferase assay. Relative promoter activities are referred to the activity of the unmutated wildtype construct, which was defined as 100%. Values are means ± SEM of six biological replicates. **p < 0.01; ***p < 0.001; ****p < 0.0001 (Mann-Whitney U-test).

Since an indirect *XYLT1* regulation through miRNA-29b was described here for the first time, we further evaluated whether Sp1 might be involved in this regulatory mechanism. Seven Sp1 binding sites (named SP1.1 to SP1.7), harboring a GC-rich binding motive, were identified in the *XYLT1* promoter region, as shown in Fig. 5. Individual deletion of each Sp1 transcription factor binding site via site-directed mutagenesis was assessed by mutating the GC-rich binding motive to an AT-rich sequence in existing *XYLT1* promoter coding luciferase vectors, as has been described previously^32^. SW1353 cells were transfected with the appropriate *XYLT1* promoter construct and a dual luciferase assay was performed to analyze the influence of Sp1 transcription factor binding site deletion. Relative luciferase activity of diverse promoter constructs was compared with *XYLT1* promoter activity of the wildtype promoter sequence. Each mutation of a Sp1 transcription factor binding site resulted in a decrease in relative promoter activity, as illustrated in Fig. 6. Promoter activity decreased significantly from 100% (wildtype construct) to 24.6% (SP1.1), 42.2% (SP1.3) and 54.2% (SP1.5), respectively. In summary, these results underline the relevance of Sp1 for the induction of *XYLT1* expression.

### Sp1/Sp3 transcription factor binding sites in the *XYLT1* promoter are occupied *in vitro*

**Figure 7:**
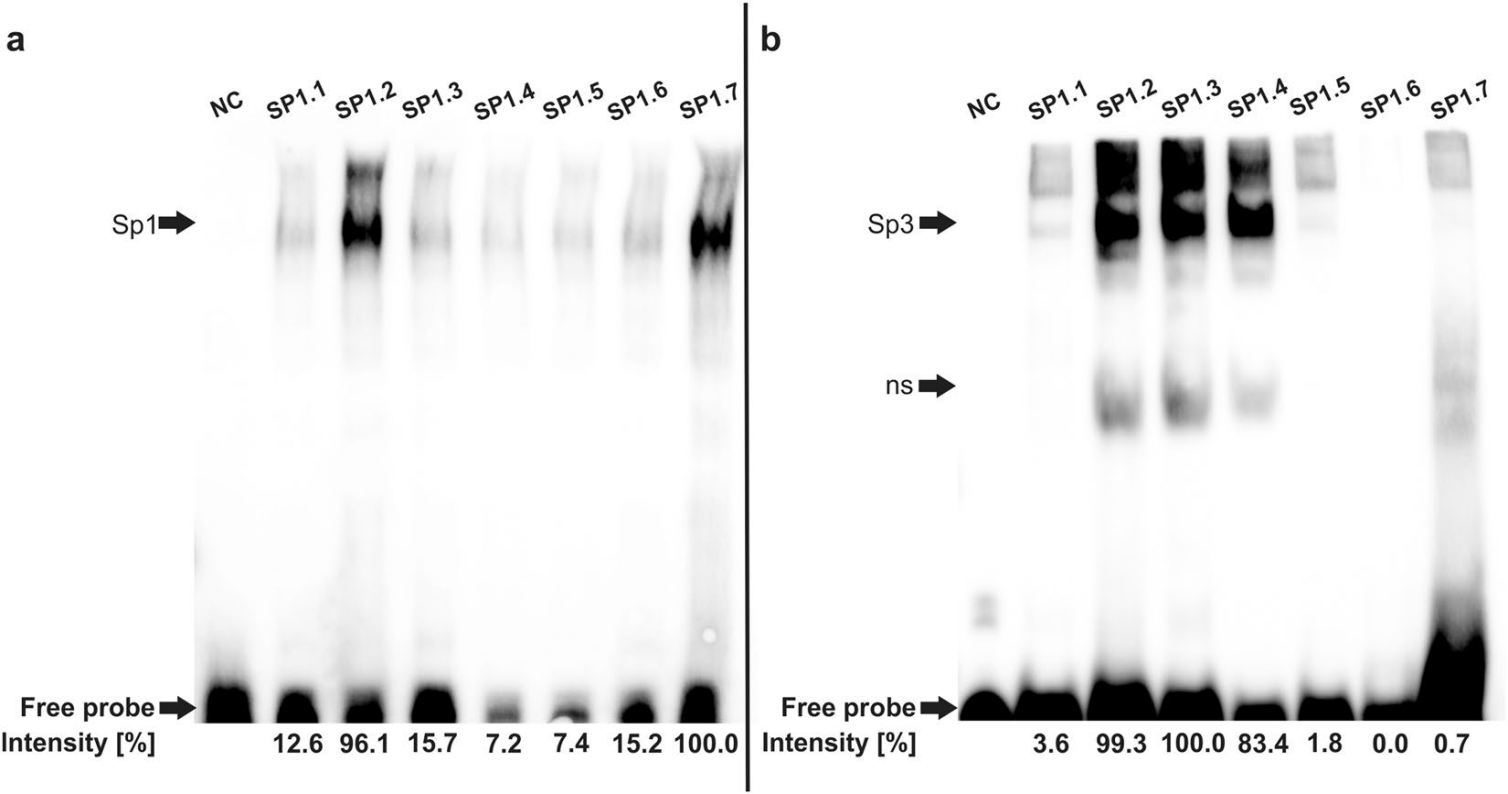
Results of the electrophoretic mobility shift assay depicting Sp1 and Sp3 binding to Sp1 transcription factor binding sites identified *in silico* in the *XYLT1* promoter region. Biotin-labeled DNA probes corresponding to Sp1 transcription factor binding sites SP1.1 to SP1.7 were incubated with recombinant Sp1 (**a**) or Sp3 (**b**) protein and analyzed on two independent gels (separated by the black line). In each line 1 (NC), an unlabeled DNA probe was used to demonstrate specificity of DNA-protein binding. Sp1 or Sp3 binding to the appropriate DNA probes was relatively quantified by using ImageJ while only a comparison between samples on one gel was performed. The strongest band in (**a**) or (**b**) represents the strongest binding of DNA to protein and was defined as 100%. ns: unspecific binding. Full-length gels (with total free probe band) are presented in Supplementary Figs S1 and S2.

After evaluating the importance of Sp1 transcription factor binding sites for basal *XYLT1* promoter activity, it was finally necessary to prove the occupation of the transcription factor binding sites by Sp1 protein. Since the Sp1 protein reveals high similarity with transcription factor Sp3, a shared occupancy of Sp1 binding sites with Sp1 and Sp3 is discussed^33^. An EMSA using a Sp1 or Sp3 protein was conducted to monitor not only Sp1, but also Sp3 binding to Sp1 binding sites in the *XYLT1* promoter. Sp1 binds to all Sp1 binding sites of the *XYLT1* promoter region, while the strongest binding was detected using an oligonucleotide probe resembling binding site SP1.2 (binding intensity 96.1%) or SP1.7 (binding intensity 100%), as depicted in Fig. 7. Contrarily, Sp3 bound strongly to binding sites SP1.2 (binding intensity 99.3%), Sp1.3 (binding intensity 100%) and Sp1.4 (binding intensity (83.4%). The remaining binding sites were not or only negligibly occupied by Sp3.

## Discussion

Fibrotic remodeling is characterized by ECM accumulation, tissue stiffening and progressive failure of the organs affected^8,34^. It has recently been investigated that fibrosis is not only associated with elevated TGF-β1 signaling, but also with aberrantly expressed miRNAs. miRNA-21, miRNA-29b and miRNA-145 are specifically suggested to play a crucial role in regulating fibrotic targets^7,35^.

Many studies are searching for suitable cellular points of application because no anti-fibrotic therapy has been established yet^36^. One of these putative targets is XT-I. Two XT isoforms, XT-I and XT-II, catalyze the rate-limiting step in PG glycosylation in humans^19,20,37^. Although biochemical studies revealed a strong functional similarity of both enzymes, TGF-β1 only induces *XYLT1* mRNA expression^38^. Increased *XYLT1* mRNA expression, XT activity and the resulting PG accumulation are associated with liver fibrosis^23^, cardiac fibrosis^38^, lung fibrosis^39^ and skin fibrosis^24,25^. Although many studies describe the involvement of miRNAs in pathobiochemistry, XT regulation by miRNAs has only been sparsely discussed so far. Theis *et al*.^40^ recently described an improvement of spinal cord injury recovery after miRNA-133b-mediated *XYLT1* downregulation. Furthermore, altered *XYLT1* expression was associated with abnormal circulating miRNA levels in patients suffering from pediatric astrocytomas^41^.

However, we report here on the first study dealing with miRNA-dependent regulation of XT-I in fibrosis – the most important disorder XT-I has been associated with. Only increasing knowledge about the regulation of fibrotic mediators, such as XT, might allow us to develop an anti-fibrotic treatment. Here, we focus on XT regulation via miRNA-29b, a miRNA which is aberrantly expressed in fibrosis of the kidney^13^, heart^14^, lung^15^ and liver^26^. miRNA-29b is a member of the highly conserved miRNA-29 family, which consists of three members: miRNA-29a, -29b and -29c, sharing an identical seed sequence^13^. Published data suggest a TGF-β1/SMAD3-mediated miRNA-29b suppression in fibrotic tissues. miRNA-29b is extensively discussed as a potential therapeutic target to heal fibrosis. Li *et al*.^42^ recently reported on the successful inhibition of endometrial fibrosis by using a miRNA-29 mimic in rats. In addition, the first clinical trial phase I (NCT02603224) using a cholesterol-conjugated miRNA-29-duplex was finished in 2017 and supports further investigation of miRNA-29b mimics as novel antifibrotic tools. We investigated for the first time whether miRNA-29b might not only be involved in fibrotic remodeling targeting collagens or fibrillin^16,28^, but also exert influence on XT-I expression. To examine this issue, we recently established a myofibroblast culture model, which is based on a low cellular density and supplementation with TGF-β1^25,43^. TGF-β1 is the key mediator of fibrotic remodeling and could be demonstrated to induce XT activity during myofibroblast differentiation^25^. Unfortunately, several trials to inhibit TGF-β1 in an antifibrotic manner revealed that TGF-β1 is involved in too many orchestrating signal cascades and, consequently, its inhibition cannot be realized^44^.

Here, we describe a TGF-β1-induced downregulation of miRNA-29b expression in our cell culture model by using a PCR array. These PCR arrays represent a great tool for evaluating expression profiles, but it is controversial to quantify miRNA expression with a SYBR green-based assay due to methodical imprecision. Therefore, we confirmed our finding with a more sensitive Taqman-based assay^45^. TGF-β1-induced suppression of miRNA-29b expression has been reported previously^46^.

There are two ways in which a miRNA can exert influence on a target mRNA. On the one hand, the miRNA can bind directly to the 3′UTR, provoking mRNA decay or translational inhibition. On the other hand, indirect regulation via targeting a signaling molecule further targeting one or several mRNAs might be involved^2^. We could demonstrate here that *XYLT1* is neither a target of miRNA-29b identified *in silico* nor an experimentally confirmed 3′UTR binding partner of miRNA-29b. Therefore, we can exclude a direct targeting of *XYLT1* 3′UTR by miRNA-29b. However, by transfecting NHDF with the miRNA-29b inhibitor resembling fibrosis, we revealed for the first time a strong induction of *XYLT1* mRNA expression, which was verified by quantifying the XT activity. This important finding qualifies us to hypothesize that miRNA-29b mediates indirect XT-I regulation via one or several hitherto undescribed pathway(s) and mediator(s).

We and others have depicted that Sp1 is a direct target of miRNA-29b. Sp1 is a transcription factor expressed ubiquitously which harbors three zinc-finger DNA-binding motives and regulates the transcriptional intensity of many genes, especially housekeeping genes and genes with a TATA-less GC-rich promoter region, such as *XYLT1* or *XYLT2*^32,47^. Growing evidence reveals that Sp1 is not only a transcription factor which binds GC-rich motives and is involved in maintaining basal promoter activity, but also a key regulator of fibrotic remodeling^48–50^. Binding sites for the Sp1 family were identified in the promoter region of many ECM-coding genes and blocking of Sp1 inhibits ECM gene expression broadly *in vitro* and *in vivo*^31^. However, Sp1 binding can exert a repressive or inductive effect on promoter activity in response to physiologic and pathological stimuli^47^.

The *SP1* mRNA binding score calculated was weaker than that of, for instance, *COL1A1*. This low target prediction score might indicate a weak binding. However, several studies have already examined and approved Sp1 regulation by miRNA-29b^51,52^. Müller *et al*.^30^ have pointed out previously that Sp1, in addition to the Ap1 family, drives *XYLT1* expression. They quantified a decrease of *XYLT1* mRNA expression after exposure of SW1353 cells to mithramycin, which prevents the binding of transcription factors to GC-rich DNA regions. These results were confirmed by Ye *et al*.^53^, who were able to reduce basal *XYLT1* mRNA expression via treatment with Sp1 inhibitors, mithramycin supplementation or stable Sp1 knockdown in rat nucleus pulposus cells. These studies, therefore, underline the functional interaction of XT-I and Sp1 and give a hint regarding XT-I regulation via miRNA-29b and Sp1. We exposed NHDF to miRNA-29b or its inhibitor in parallel with TGF-β1 to verify our hypothesis of Sp1 involvement in miRNA-29b-mediated XT induction. We determined a TGF-β1-mediated XT induction, which has also been described previously^25^, which was significantly gradable with supplementation of a miRNA-29b inhibitor. This result approves our hypothesis of XT induction by miRNA-29b reduction. Interestingly, *XYLT2* was regulated neither by TGF-β1 nor by miRNA-29b or its inhibitor. The lack of influence of TGF-β1 on *XYLT2* has been described previously^38^.

The increase in *COL1A1* mRNA expression observed after TGF-β1 treatment is in line with collagen accumulation in fibrotic tissues^34^ and underlines the functionality of our cell culture system. An additional cellular treatment with the miRNA-29b inhibitor resulted in a weak elevation of *COL1A1* mRNA expression, while additional exposure to miRNA-29b entailed a strong diminishment of mRNA expression. Maurer *et al*.^16^ also describe a downregulation of *COL1A1* mRNA expression after exposing SSc fibroblasts to miRNA-29a/b. This effect is attributed to the direct binding of miRNA-29 to the *COL1A1* 3′UTR. In line with our results, several published studies assess stronger effects in *COL1A1* mRNA downregulation after miRNA-29 treatment than *COL1A1* mRNA increase after miRNA-29b inhibitor treatment^16^. This might be due to the lack of a direct one-to-one stoichiometric relationship between the levels of miRNA and target gene expression, which was discussed by Li *et al*.^46^ who studied Sp1-mediated regulation of *COL1A1* in the fibroblasts of a human tenon. While miRNA-29b entails mRNA degradation directly, a miRNA-29b knockdown, by using its inhibitor, only prevents mRNA degradation by miRNA-29b but does not increase the expression obviously. Therefore, independent of cell type and molecular environment, there is a multitude of other miRNAs or mediators that might exert influence on the *COL1A1* mRNA expression intensity^46^.

Sp1 is known to be upregulated by TGF-β1. Nevertheless, apart from our observations, there are several studies which describe only a weak or no Sp1 induction after TGF-β1 treatment. Ogawa *et al*.^51^ demonstrate that miRNA-29b-induced *COL1A1* suppression is mediated by its direct interaction with *COL1A1* 3′UTR and by its interference with Sp1 expression in human stellate cells. Notably, in this context, only a weak *SP1* mRNA and protein expression reduction by miRNA-29b was detected, while TGF-β1 treatment did not exert influence on *SP1* mRNA or protein expression. Contrarily, Sp1 protein is strongly increased in tubular epithelial cells after TGF-β1 supplementation^13^. Therefore, TGF-β1-mediated Sp1 induction seems to be generally weak and dependent on the cellular system. Vanden Oever *et al*.^54^ recently described type VII collagen downregulation in recessive dystrophic epidermolysis bullosa via miRNA-29b-mediated Sp1 repression. Comparable to the results gained here, *SP1* mRNA expression was downregulated in a small but statistically significant extent by miRNA-29b transfection in dermal fibroblasts. In our study, the Sp1 protein level was not diminished by treating the cells with miRNA-29b. Contrarily, the Sp1 protein expression was significantly increased after combined TGF-β1 and miRNA-29b inhibitor treatment.

**Figure 8:**
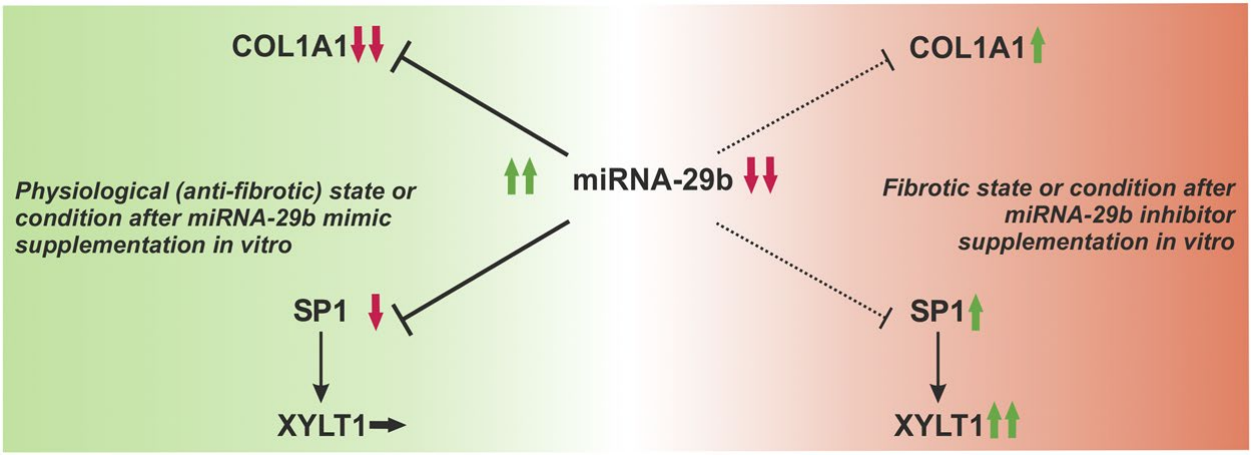
Hypothetical model of *XYLT1* regulation via miRNA-29b. Physiologically, miRNA-29b suppresses the expression levels of *COL1A1* and *SP1*, which are direct targets of the miRNA. Suppression of *COL1A1* and *SP1* goes along with anti-fibrotic remodeling, which is also achieved by using miRNA-29b mimics *in vitro*. Sp1 is known to stimulate *XYLT1* mRNA expression, but the *XYLT1* expression might not be affected by an SP1 decrease due to the availability of several other influencing transcription factors and mediators (left side). The miRNA-29b level is diminished in fibrosis. This state is reproducible by using miRNA-29b inhibitors *in vitro*. A decrease in miRNA-29b implies a minor inhibition of *COL1A1* and *SP1* mRNA expression, which can contribute to *COL1A1* and *SP1* upregulation. Sp1 upregulation provokes a *XYLT1* induction (right side). Aside from *XYLT1* regulation via Sp1, additional mechanisms might exist which have not yet been elucidated.

Thus, it can be hypothesized that Sp1 mediates a multitude of signaling pathways, although it is often only weakly regulated – called fine-tuning by Jiang *et al*.^13^. In summary, the results described here reveal indirect XT regulation via miRNA-29b and mention Sp1 as the putative missing link (Fig. 8). In order to determine this issue, we deleted Sp1 transcription factor binding sites and evaluated the influence on *XYLT1* promoter activity, on the one hand, and proved the affinity of Sp1 protein to the binding sites identified *in silico*, on the other hand. We published the first successful amplification and sequencing of the complete *XYLT1* promoter region in 2014^29^ and pointed out that the *XYLT1* human reference sequence had been incomplete until then. Here, we identified seven Sp1 transcription factor binding sites in the *XYLT1* promoter region *in silico* and demonstrated *in vitro* that they are occupied. Deletion by site-directed mutagenesis indicated that every mutated binding site resulted in a diminished *XYLT1* promoter activity. Different intensities of downregulation might possibly arise from the synergistic interaction of several transcription factors in response to the appropriate binding site. This phenomenon has already been shown concerning the *XYLT2* promoter^32^. Khair *et al*.^55^ also performed mutation experiments of Sp1 binding sites in the *XYLT1* promoter region. In contrast to our results, they only identified two binding sites and detected opposing effects of these binding sites on basal *XYLT1* promoter activity. Differences in both studies might result from the reference sequence considered. Summing up, it was pointed out that Sp1 is an activating transcription factor for XT-I, which is involved in XT regulation via miRNA-29b, according to our hypothesis.

Surprisingly, it was assessed that a Sp1 knockdown did not exert influence on *XYLT1* mRNA expression in SW1353 cells. We confirmed this finding because miRNA-29b-mediated Sp1 knockdown did not influence *XYLT1* mRNA expression in NHDF. However, Ye *et al*.^53^ observed a strong diminishment of *XYLT1* mRNA expression after stable Sp1 knockdown. Thus, Sp1-mediated XT regulation might depend on the cellular context. It is probable that a Sp1 knockdown leads to the recruitment and occupancy of Sp1 transcription factor binding sites by Sp3. Binding of Sp3 to several Sp1 binding sites, for example, in the *XYLT1* promoter, has been shown here and elsewhere^33^. Concerning the regulatory potential of Sp1, it is expected that not only the cellular context and posttranslational Sp1 modifications, but also the molecular ratio of Sp1 to Sp3 play an important role. Additionally, Sp1 is known to interact with various other transcription factors, such as SMAD^56^, c-myc^57^ or c-jun^58^. Conflicting knowledge about the influence of Sp1 and Sp3 on *XYLT1* promoter activity also emerges when looking at Sp3-mediated *XYLT1* regulation. While Müller *et al*.^30^ and Ye *et al*.^53^ describe a decrease of *XYLT1* mRNA expression indicating a positive effect on the expression level after Sp3 knockdown, Khair *et al*.^55^ propose a repressive function of Sp3 on the *XYLT1* promoter in human primary chondrocytes. Hence, *XYLT1* expression is influenced by Sp1 and Sp3 levels, which are, in turn, dependent on variable cellular environmental conditions.

In summary, our data suggest that miRNA-29b influences fibrotic induction of human XT-I through the Sp1 signaling pathway. This new finding does not only deepen our insights into pathobiochemical signaling in fibrosis, but also encourages us to develop targeted anti-fibrotic strategies in the future. Further studies will evaluate whether there are additional molecules, apart from Sp1, which mediate XT induction in fibrosis.

## Materials and Methods

### Fibroblast cultivation and treatment

Normal human dermal fibroblasts were purchased from Genlantis (San Diego, USA) and Coriell (Camden, USA). Unless otherwise stated, the NHDF were cultivated at 37 °C in a humidified atmosphere of 5% CO_2_ in Dulbecco’s modified Eagle’s medium (DMEM; Thermo Fisher Scientific, San Diego, USA) supplemented with 10% fetal calf serum (FCS; VWR, Radnor, USA), 1% antibiotic/antimycotic solution (PAN Biotech, Aidenbach, Germany) and 2% L-glutamine (PAN Biotech, Aidenbach, Germany). Cell culture medium was replaced twice a week and after reaching confluence, cells were expanded or used for experimental settings.

Cells were seeded (50 cells/mm^2^) and incubated for 24 h to evaluate the influence of TGF-β1 on the expression of miRNAs. Thereafter, cells were washed with phosphate-buffered saline (PBS; Thermo Fisher, San Diego, USA) and a serum withdrawal to 0.1% FCS was performed. After another 24 h, cell culture medium was replaced by medium containing 0.1% FCS and 5 ng/mL TGF-β1 (Miltenyi, Bergisch Gladbach, Germany) or PBS, respectively. Cell harvesting was executed after 48 h and cell lysates were stored at −80 °C. All treatments were performed in biological triplicates.

miRNA-29b inhibitor (UAGCACCAUUUGAAAUCAGUGUU, Thermo Fisher Scientific, San Diego, USA) or a negative control miRNA (final concentration of each miRNA: 50 nM) were transfected into NHDF using Lipofectamine 2000 (Thermo Fisher, San Diego, USA). The NHDF were seeded (50 cells/mm^2^) in DMEM without antibiotic/antimycotic solution in the intervening time of preparing transfection reactions. Cell culture medium was replaced 24 h after transfection. All treatments were performed in biological triplicates and transfection efficiency was determined using a FAM-labeled negative control siRNA (Thermo Fisher, San Diego, USA). Cells were harvested after 48 or 144 h and stored at −80 °C, while cell culture supernatants were stored at −20 °C.

Cells were seeded (50 cells/mm^2^) to analyze the effects of miRNAs under supplementation of TGF-β1. Cell culture medium was replaced with DMEM without antibiotic/antimycotic solution after 24 h. Transfection reactions (final concentration of each miRNA: 50 nM; miRNA-29b AACACUGAUUUCAAAUGGUGCUA) were prepared and added to the cells. Cell culture medium was replaced with DMEM containing PBS or TGF-β1 (5 ng/mL), respectively, 6 h post-transfection. Each treatment was seeded in biological triplicates and cells were harvested after 24 or 144 h. Cell lysates intended for gene expression analyses or Sp1 ELISA were stored at −80 °C.

### Nucleic acid extraction

Cell lysates for quantification of miRNA expression via miScript miRNA HC polymerase chain reaction (PCR) array Human miFinder 384HC (Qiagen, Hilden, Germany) or TaqMan advanced miRNA assays (Thermo Fisher, San Diego, USA) were lysed in QIAzol Lysis Reagent. Biological replicates of lysates for the array were pooled. Total RNA was extracted according to the manufacturer’s description of the miRNeasy Micro Kit (Qiagen, Hilden, Germany).

Contrarily, total RNA extraction out of NHDF cell lysates to quantify mRNA expression levels via SYBR green-based real-time PCR was performed according to the manufacturer’s instructions using the NucleoSpin RNA Kit (Macherey-Nagel, Düren, Germany). Harvested cells were lysed in RA1 buffer and processed. An amount of 50 µL of the cell lysates was separated to quantify the total DNA content using the NucleoSpin Blood Kit (Macherey-Nagel, Düren, Germany). Nucleic acid concentrations were quantified using the NanoDrop 200 spectral photometer (Peqlab, Erlangen, Germany).

### miRNA and mRNA expression analyses

A gene expression profile of 372 miRNAs was performed via quantitative real-time PCR using the miScript miRNA PCR Array Human miFinder 384HC (Qiagen, Hilden, Germany). After total RNA extraction, miRNAs were reverse transcribed according to the manufacturer’s instructions of the miScript II RT Kit (Qiagen, Hilden, Germany). As instructed by the manufacturer, a PCR mastermix was prepared using the miScript SYBR Green PCR Kit (Qiagen, Hilden, Germany) and added to the 384-well PCR array plate to be cycled as indicated. Data analyses of 372 target genes were calculated with a free data analysis software (miScript miRNA PCR Array Data Analysis, Qiagen, Hilden, Germany) using the delta-delta CT method.

Complementary to the PCR array, miRNA-29b expression was quantified using a Taqman probe. All reaction and incubation steps were performed according to the manufacturer’s instructions of the TaqMan Advanced miRNA cDNA Synthesis Kit (Thermo Fisher, San Diego, USA) and the TaqMan advanced miRNA Assay (Thermo Fisher, San Diego, USA). Each sample was measured in technical triplicates. miRNA-29b expression was normalized to the housekeeping gene miRNA-191 expression via the delta-delta CT method for data acquisition.

Relative quantification of mRNA expression levels with SYBR green-based real-time PCR was carried out, as has been described previously^59^. Briefly, 1 µg RNA was reverse transcribed to cDNA using the SuperScript II Reverse Transcriptase Kit (Thermo Fisher, San Diego, USA). Real-time PCRs were performed using SYBR Green Master Mix (Roche, Mannheim, Germany). Sequences of intron-spanning primers are listed in Table 2. Gene expression levels of XT-I (*XYLT1*), XT-II (*XYLT2*), collagen type 1 alpha 1 (*COL1A1*) and specificity protein 1 (*SP1*) were referred to a normalization factor calculated from mRNA expression levels of three housekeeping genes (β2-microglobuline (*B2M*), hypoxanthine phosphoribosyltransferase 1 (*HPRT1*) and glyceraldehyde-3-phosphate dehydrogenase (*GAPDH*)). All samples were analyzed in technical triplicates.

**Table 2:** List of PCR systems for quantitative real-time PCR.

Gene	Protein	5′-3′-sequence	T_OA_ [°C]
*hB2M*	B2M	TGTGCTCGCGCTACTCTCTCTT CGGATGGATGAAACCCAGACA	63.0
*hCOL1A1*	COL1A1	GATGTGCCACTCTGACT GGGTTCTTGCTGATG	63.0
*hGAPDH*	GAPDH	AGGTCGGAGTCAACGGAT TCCTGGAAGATGGTGATG	63.0
*hHPRT*	HPRT	GCTGACCTGCTGGATTAC TGCGACCTTGACCATCTT	63.0
*hSP1*	Sp1	GAGCTACAGAGGCACAAACG CCTGGGCCTCCCTTCTTATTC	63.0
*hXYLT1*	XT-I	GAAGCCGTGGTGAATCAG CGGTCAGCAAGGAAGTAG	63.0
*hXYLT2*	XT-II	ACACAGATGACCCGCTTGTGG TTGGTGACCCGCAGGTTGTTG	63.0

### XT activity assay

Quantification of XT activity in cell culture supernatants has been described previously^60,61^. Taken together, the method relies on incorporation of the donor UDP-[^14^C]-xylose (Perkin Elmer, Foster City, USA) into silk fibroin protein acceptor. Quantified disintegrations per minute (dpm) reflect the XT activity, because the latter is proportional to UDP-[^14^C]-xylose incorporation. Each biological sample was measured in technical duplicates and referred to the total DNA content of the appropriate cell lysate.

### Quantification of the SP1 protein expression by enzyme-linked immunosorbent assay

The Sp1 protein content in NHDF lysates was quantified using a commercially available ELISA kit (Cloud-Clone Corp., Katy, USA). Isolation of nuclear proteins was performed using the NE-PER Nuclear and Cytoplasmic Extraction Reagent (Thermo Fisher, San Diego, USA), according to the manufacturer’s protocol. Absolut Sp1 protein amount was referred to total nuclear protein content. The experiment was performed twice and there was one technical replicate for each biological sample.

### Verification of miRNA-29b binding to the 3′ untranslated region of *XYLT1*

Human chondrosarcoma SW1353 cells, purchased from ATCC (Manassas, USA), were seeded in a cell density of 180,000 cells/6-well to analyze the potential direct binding of miRNA-29b to the *XYLT1* 3′UTR. Unless otherwise stated, SW1353 cells were cultivated at 37 °C in a humidified atmosphere of 5% CO_2_ in RPMI 1640 medium containing 10% FCS and 1% antibiotic/antimycotic solution. The cell culture medium was replaced twice a week and, after reaching confluence, the cells were expanded or used for experimental settings. After 24 h, the cell culture medium was exchanged for medium without antibiotic/antimycotic solution. A negative control miRNA or miRNA-29b (final concentration 50 nM) and 50 ng of *XYLT1*-3′UTR coding plasmids (HmiT016805a-MT06/HmiT016805b-MT06; GeneCopoeia, Rockville, USA) was transfected into the cells using Fugene 6 (Promega, Mannheim, Germany). Cell culture medium was replaced 24 h later. The cells were lysed in lysis buffer, according to the manufacturer’s instructions of the Luc-Pair Duo-Luciferase Assay Kit 2.0 (GeneCopoeia, Rockville, USA) 48 h after transfection and stored at −80 °C. Each transfection reaction was performed in six biological replicates. Quantification of the constitutive renilla luciferase activity and *XYLT1*-3′UTR-regulated firefly luciferase activity was performed on a Tecan Reader infinite 200 Pro (Tecan Group, Männedorf, Swiss) and firefly luciferase activity was normalized to renilla luciferase activity.

### Site-directed mutagenesis of Sp1 transcription factor binding sites in the *XYLT1* promoter region

Cloning of the luciferase reporter vector pGL4.10 *XYLT1* c. − 1639 to c. + 1_complete_ containing the complete *XYLT1* promoter region has been described previously^29^. Site-directed mutagenesis of the Sp1 transcription factor binding sites identified was performed using the QuikChange II XL Site Directed Mutagenesis Kit (Agilent, Santa Clara, USA), according to the manufacturer’s instructions. Four to six bases within an identified Sp1 transcription factor binding site were mutated to an adenine/thymine-rich DNA sequence, so that the Sp1 binding site disappeared. Successful mutations were verified by direct plasmid sequencing, as has been described previously^29^. Sequences of mutation and sequencing primers used are listed in Table 3.

**Table 3:** List of site-directed mutagenesis (SDM) and sequencing primer sequences. T_OA_: optimal annealing temperature.

Primersite	5′-3′-sequence	T_OA_ [°C]
**Site-directed mutagenesis**		
***SP1.1***	TCACCCAAAGCCTCCTCCC**TTAAG**CTTCAAGCCCAGGATGAA TTCATCCTGGGCTTGAAG**CTTAA**GGGAGGAGGCTTTGGGTGA	60.0
***SP1.2***	TGTCTTCTCTGATCGCCCC**TTAA**CCCTTCCTTTCCCTCCTCC GGAGGAGGGAAAGGAAGGG**TTAA**GGGGCGATCAGAGAAGACA	60.0
***SP1.3***	TCCTTTTCCTCCCTCGGC**CTTAAG**CCCAGGCCCCGCCCTCTC GAGAGGGCGGGGCCTGGG**CTTAAG**GCCGAGGGAGGAAAAGG	60.0
***SP1.4***	CGGCTCCTCCCCCAGGCCC**TTAAG**TCTCCGCCTCGGCCCGCG CGCGGGCCGAGGCGGAGA**CTTAA**GGGCCTGGGGGAGGAGCCG	68.0
***SP1.5***	CAGCGGGGACAGGGGTGTG**AATTC**GGGGCGCCGCGCGGGCCA TGGCCCGCGCGGCGCCCC**GAATT**CACACCCCTGTCCCCGCTG	68.0
***SP1.6***	GGCGCCGCGCGGGCCAGGC**TTAAA**CCCCTCCCTGCGCGCCCC GGGGCGCGCAGGGAGGGG**TTTAA**GCCTGGCCCGCGCGGCGCC	68.0
***SP1.7***	GGCCAGGCGCCCGCCCCTC**TTAAG**GCGCCCCGTCCCCGAGCG CGCTCGGGGACGGGGCGC**CTTAA**GAGGGGCGGGCGCCTGGCC	68.0
**Sequencing**		
***XYLT1*** **_A**	CCCTGTTTCGCGGCCCCTG	58.8
***XYLT1*** **_B**	CCCCATCCTACTCCCACTAC	58.8
***XYLT1*** **_C**	GAAGGAAAGGGAGGAGGAAA	58.8
**pGL4.10**	CTTAATGTTTTTGGCATCTTCCA	58.8

### Quantification of *XYLT1* promoter activity

As indicated above, SW1353 cells were seeded and incubated overnight. After 24 h, 1 µg of mutated firefly luciferase coding pGl4.10 plasmid and 10 ng of renilla luciferase coding pGl4.74 control plasmid were transfected into the cells using Fugene 6^29^. Each of the mutated plasmids was amplified and, finally, transfected in six biological replicates. The medium was replaced after 24 h and cell harvesting was performed after a total of 48 h. Cell lysis and quantification of luciferase activity were conducted using the Dual Luciferase Reporter Assay System (Promega, Mannheim, Germany), as has been described previously, according to the manufacturer’s instructions^29^. Relative luciferase activity, resembling *XYLT1* promoter activity, was calculated as the quotient of firefly/renilla luciferase activity of each sample.

### Electrophoretic mobility shift assay (EMSA)

The binding of Sp1 or Sp3 to the transcription factor binding sites identified *in silico* was confirmed with 5′-biotinylated oligonucleotides (sequences listed in Table 4) which contained the putative Sp1 transcription factor binding sites of the *XYLT1* promoter. Detection of the Sp1/3 DNA-protein complexes was performed using the LightShift chemiluminescent EMSA kit (Thermo Fisher, San Diego, USA). An amount of 1 μg recombinant Sp1 protein (OriGene, Rockville, USA) was incubated in a buffer containing 10 mM Tris, 50 mM KCl, 1 mM DTT (pH 7.5, binding buffer), 75 ng/µl poly(dI⋅dC), 0.25 mg/ml BSA and 40 fmol of the biotinylated, hybridized oligonucleotide. An amount of 1 μg of recombinant Sp3 protein (OriGene, Rockville, USA) was incubated in the same buffer to which was added 5% glycerol, 5 mM MgCl_2_ and 0.025% NP-40. The reaction mixture was left to incubate for 20 min at room temperature. Finally, the DNA-protein complexes were mixed with loading buffer and resolved on 6% polyacrylamide gel (Thermo Fisher, San Diego, USA) in 0.5x TBE buffer for 1.5 h at 100 V after a pre-run of the gel for 1 h at 100 V. The DNA-protein complexes were transferred to a positively charged nylon membrane (380 mA, 30 min) in 0.5x TBE and crosslinked to the membrane by ultraviolet light (312 nm) for 10 min. The following steps were performed according to the manufacturer’s protocol. Finally, DNA-protein complexes were detected with a transilluminator.

**Table 4:** List of EMSA probes (5′-biotinylated).

Probe	5′-3′-sequence
***SP1.1***	Biotin-GCCTCCTCCCCACCCCTTCAAGCCC Biotin-GGGCTTGAAGGGGTGGGGAGGAGGC
***SP1.2***	Biotin-CTGATCGCCCCCACCCCCTTCCTTT Biotin-AAAGGAAGGGGGTGGGGGCGATCAG
***SP1.3***	Biotin-TCCTCCCTCGGCTCCTCCCCCAGGC Biotin-GCCTGGGGGAGGAGCCGAGGGAGGA
***SP1.4***	Biotin-CCCAGGCCCCGCCCTCTCCGCCTCG Biotin-CGAGGCGGAGAGGGCGGGGCCTGGG
***SP1.5***	Biotin-ACAGGGGTGTGGGGAGGGGGCGCCG Biotin-CGGCGCCCCCTCCCCACACCCCTGT
***SP1.6***	Biotin-GCCGCGCGGGCCAGGCGCCCGCCCC Biotin-GGGGCGGGCGCCTGGCCCGCGCGGC
***SP1.7***	Biotin-CCCCTCCCTGCGCGCCCCGTCCCCG Biotin-CGGGGACGGGGCGCGCAGGGAGGGG

### *In silico* analyses

The Sp1 binding sites firstly had to be identified before being mutated. The Genomatix online software suite MatInspector^62^ was utilized for this application and to exclude the occurrence of unintended transcription factor binding sites in the mutated DNA sequences. Target prediction analysis was performed using three software tools: PicTar^63^, TargetScan Human^64^ and DIANA microT^65^.

### Statistical analysis

All experimental data generated were processed and analyzed using the nonparametric two-tailed Mann-Whitney U Test in GraphPad Prism 5.0 (GraphPad Software Inc., La Jolla, USA). The significance level was defined as p < 0.05.

## Electronic supplementary material

Dataset 1
