# Supplementary material for: microRNA-29b mediates fibrotic induction of human xylosyltransferase-I in human dermal fibroblasts via the Sp1 pathway
Source: Sci Rep. 2018 Dec 12;8:17779. doi: 10.1038/s41598-018-36217-2 (PMC6290791; doi:10.1038/s41598-018-36217-2)

microRNA-29b mediates fibrotic induction of human xylosyltransferase-I in human dermal fibroblasts via the Sp1 pathway

Lara Riedel[a], Bastian Fischer[a], Thanh-Diep Ly[a], Doris Hendig[a], Joachim Kuhn[a], Cornelius Knabbe[a] and Isabel Faust[a*]

[a]Institut für Laboratoriums- und Transfusionsmedizin, Herz- und Diabeteszentrum Nordrhein-Westfalen, Universitätsklinik der Ruhr-Universität Bochum, Georgstraße 11, 32545 Bad Oeynhausen, Germany

[*]Corresponding author:
Dr. rer. nat. Isabel Faust
Institut für Laboratoriums- und Transfusionsmedizin
Herz- und Diabeteszentrum Nordrhein-Westfalen
Universitätsklinik der Ruhr-Universität Bochum
Georgstraße 11, 32545 Bad Oeynhausen, Germany
Tel.: (+49) 5731-97-1234, Fax: (+49) 5731-97-1959
E-mail: ifaust@hdz-nrw.de

Supplementary information

Figure S1

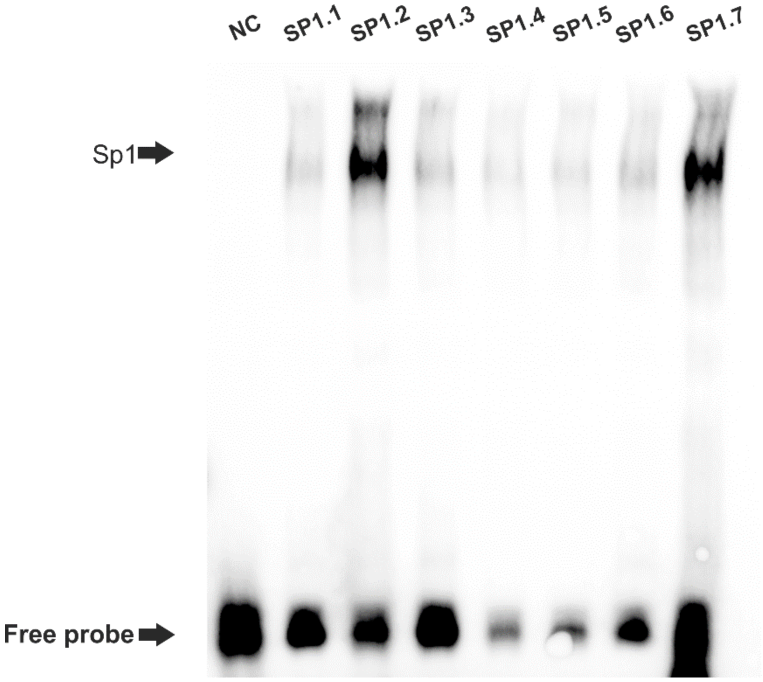

Figure S2

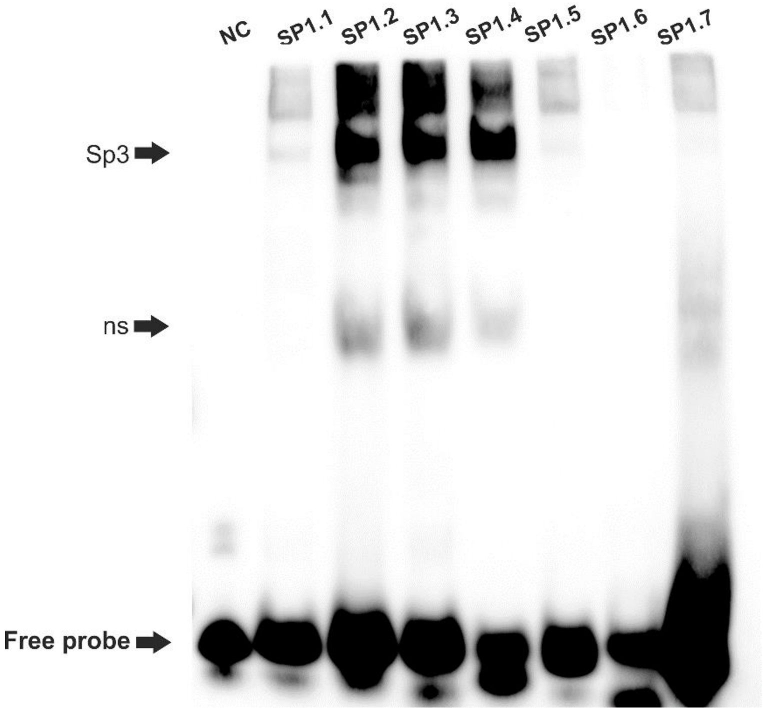

Supplement: Supplementary file 1 — Dataset 1 [file 41598_2018_36217_MOESM1_ESM.pdf]
